# Management of life‐threatening post‐traumatic otorrhagia

**DOI:** 10.1002/ccr3.3340

**Published:** 2020-12-10

**Authors:** Gabriel Rivera, Shayanne Lajud, Leonardo Gonzalez, Miguel Garratón, Eduardo Labat

**Affiliations:** ^1^ Otolaryngology‐Head and Neck Surgery University of Puerto Rico School of Medicine San Juan Puerto Rico; ^2^ Otolaryngology‐Head and Neck Surgery Department Medical University of South Carolina Columbia SC USA; ^3^ Department of Diagnostic Radiology – Neuroradiology University of Puerto Rico School of Medicine San Juan Puerto Rico

**Keywords:** acute medicine, ear, nose and throat

## Abstract

Otorrhagia can be life‐threatening, and acute control of the hemorrhage using easily accessible and practical techniques in the otolaryngology field such as Merocel packing and Kerlix gauze pressure dressing is essential to manage this complication.

## Graphical abstract

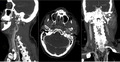

## 1 INTRODUCTION

In the setting of a traumatic injury to the temporal bone, otorrhagia, the clinical sign of bleeding per the external auditory canal (EAC), is a common occurrence. It is due to disruption of the EAC skin or the middle ear mucosa.^1^ This hemorrhage is usually self‐limited and requires no specific invasive management. Most temporal bone fractures are due to blunt trauma, but penetrating injuries may also occur.^2^ There are several documented cases of otorrhagia due to nontraumatic internal carotid artery pathology, namely, aneurysms, in the middle ear space.^3^, ^4^ However, to our knowledge, there are no documented cases of trauma to the temporal bone presenting with life‐threatening otorrhagia. In addition, due to the rarity of severe otorrhagia in general, there is no existing literature on its management. We present a penetrating gunshot wound to the temporal bone resulting in acute life‐threatening otorrhagia and propose a logical management algorithm.

## 2 CASE REPORT

**Figure 1:**
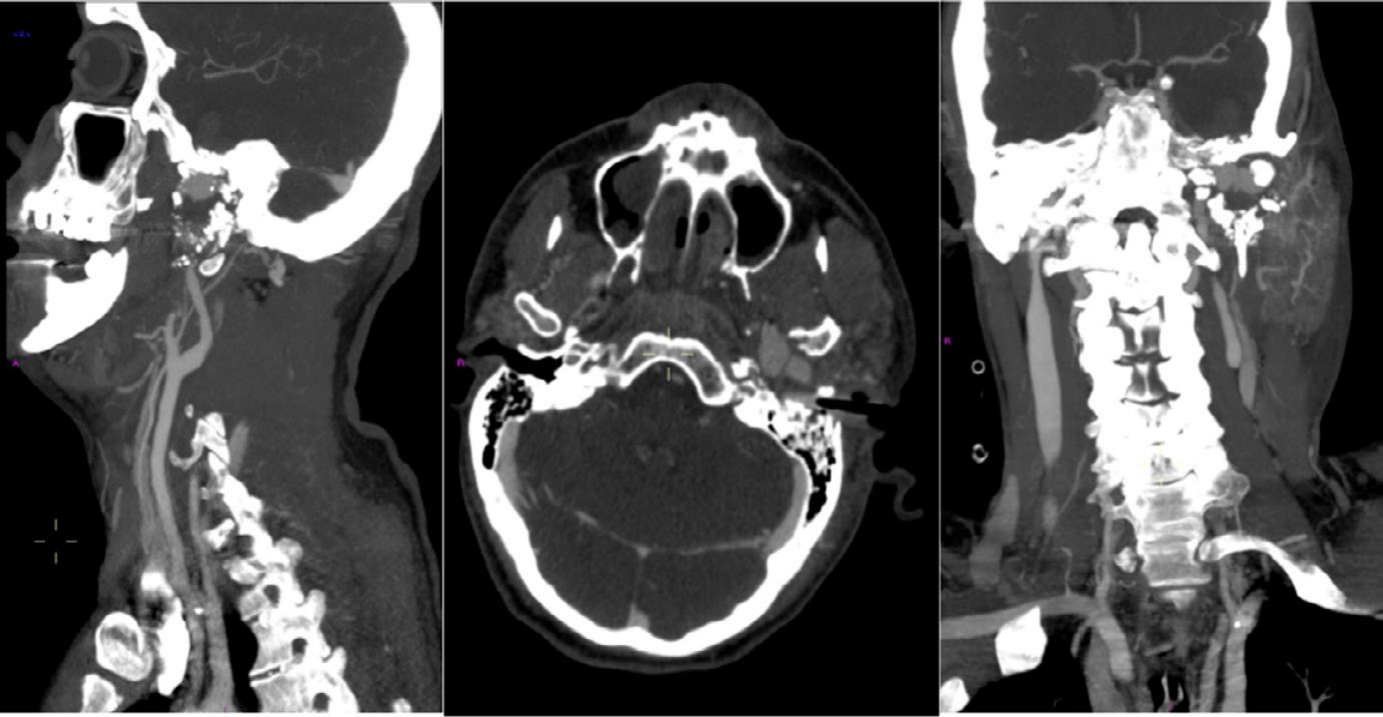
Sagittal, Axial and Coronal view of Temporal Bone CT angiography. The left ECA, left proximal middle meningeal artery, and the left pseudoaneurysm captured on key images

**Figure 2:**
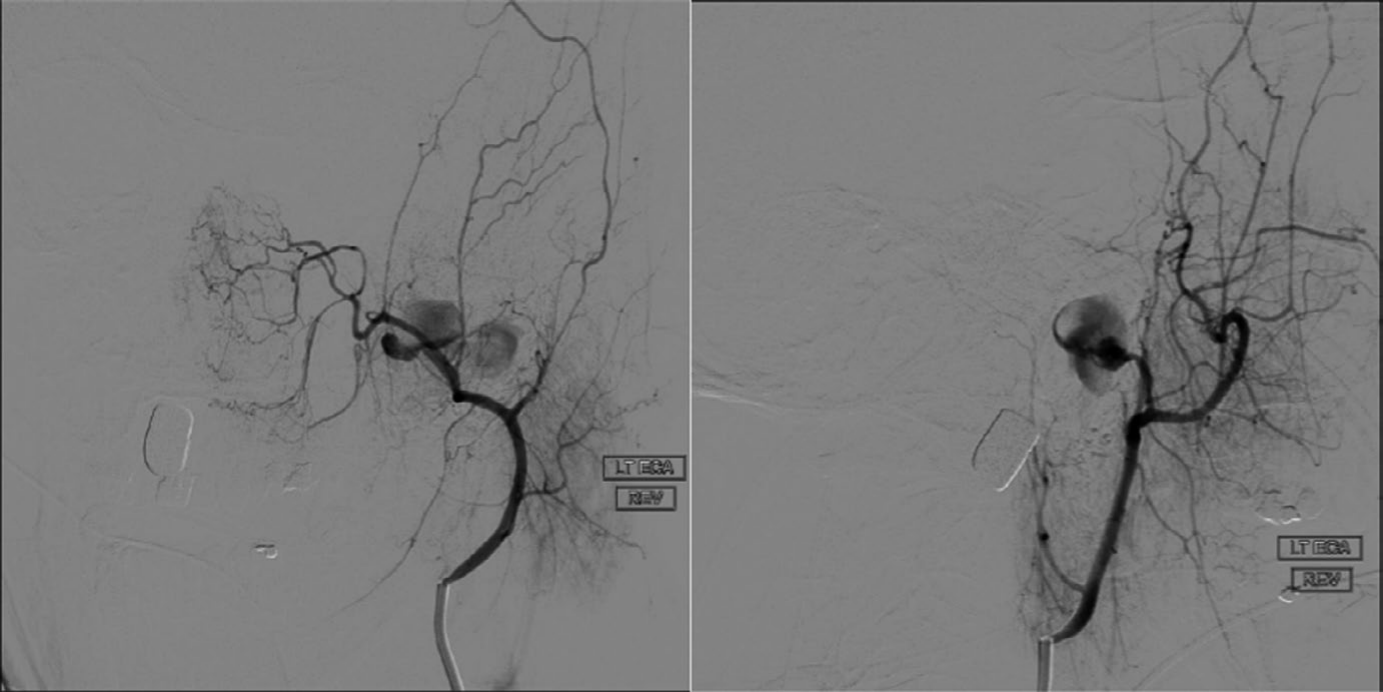
Angiography performed on the patient few minutes after episode of severe otorrhagia of the left external carotid artery pseudoaneurysm before coiling

**Figure 3:**
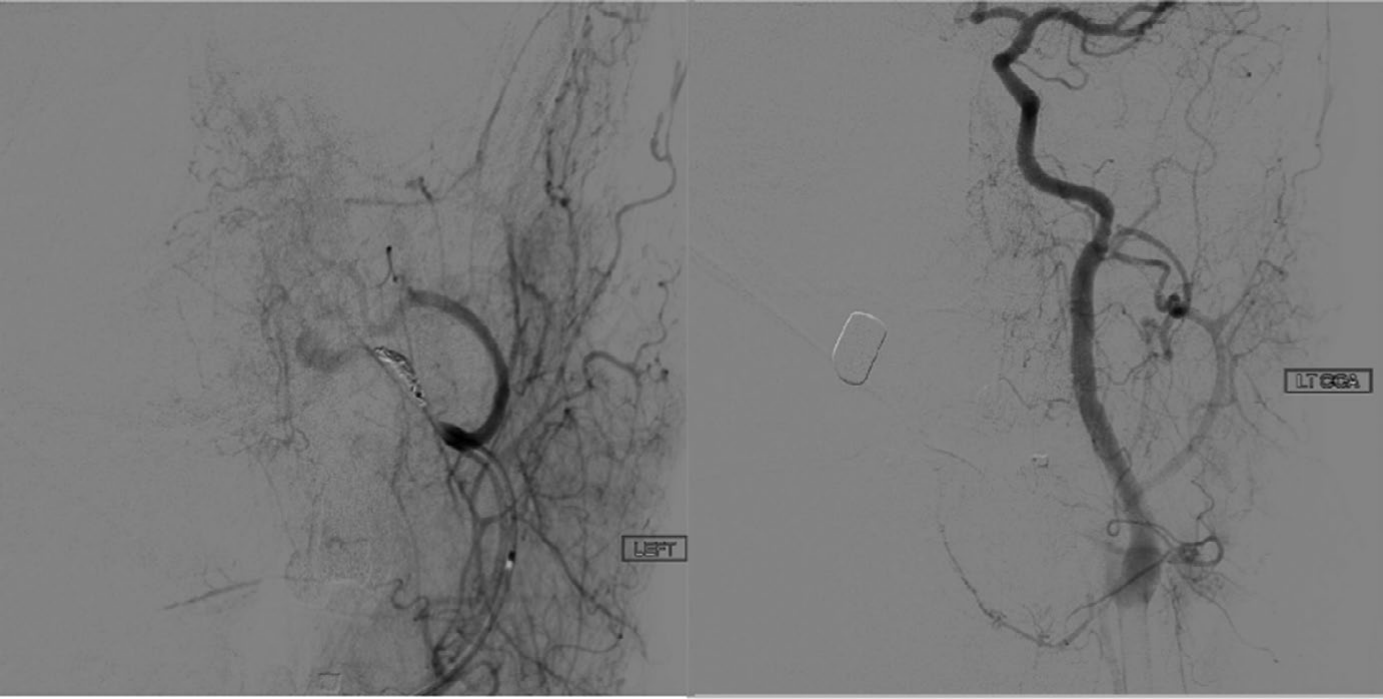
Angiography performed on the patient postcoiling with visualized obliteration of the external carotid artery pseudoaneurysm

A 39‐year‐old male presented to the emergency department after suffering a gunshot wound to the left postauricular region. A noncontrast head computed tomography (CT) showed a complex comminuted left temporal bone fracture extending to the EAC and to the carotid canal. The patient was intubated due to altered mental status. He had mild tachycardia, but otherwise normal vital signs. Otoscopic evaluation revealed abundant soft tissue collapsing within the EAC mixed with fresh bright red blood and blood clots but no active bleeding. A nonabsorbable hollow stent was placed in the lateral EAC to keep the canal patent. The patient had repeat imaging studies including a dedicated temporal bone CT and a CT angiogram (CTA) and CT venogram (CTV) of the head. Initial hemoglobin level was 11.8 g/dL. Shortly after the repeat imaging studies were performed, the nursing staff and trauma team immediately notified the otolaryngology service due to profuse uncontrollable left otorrhagia. The hollow stent was removed, and hemostasis was successfully accomplished using an anterior nasal Merocel packing followed by a mastoid dressing. A repeat CBC demonstrated a substantial drop of 2.8 g/dL of hemoglobin down to 9g/dL. The patient progressively went into hemorrhagic shock. The new imaging studies demonstrated a bullet wound coursing longitudinally through the left temporal bone with extensive destruction throughout its path, including the EAC, tympanic cavity, scutum, mastoid, and facial nerve pathway. CTA/CTV showed a lobulated arterially enhancing lesion, measuring 2.9 by 1.5 cm between the fractured and anteriorly displaced left mandibular condyle and the partially destroyed left condylar fossa; most consistent with a post‐traumatic left external carotid artery pseudoaneurysm (Figure 1). The neuroendovascular service was consulted, and the patient was rushed to their interventional radiology suite. They identified an active bleeding artery and used an Axium 3D coil to outline and obliterate the pseudoaneurysm (Figure 2). Postembolization angiography demonstrated successful control of the bleeding (Figure 3).

On postembolization day one, the patient remained critically ill. The left mastoid dressing was in place and dry, and it was removed. However, the EAC packing was kept in place for 6 days. Incomplete left eye closure was noted, suggesting facial paralysis. His hemoglobin levels dropped to 7.8 g/dL requiring multiple units of PRBCs. No further otorrhagia was noted after the embolization procedure. After removal of the EAC Merocel packing, otoscopy demonstrated circumferential abrasions with soft tissue displaced medially without clear visualization of the tympanic membrane or the ossicular chain but no active bleeding. The patient was then discharged home and unfortunately lost to follow up.

## 3 DISCUSSION

Temporal bone fractures are frequently observed in the trauma setting. However, a temporal bone penetrating wound with development of an external carotid artery pseudoaneurysm (ECA‐PA) is a rare and worrisome finding. Most pseudoaneurysms are post‐traumatic or idiopathic.^2^, ^5^ In our case, the patient developed an ECA‐PA after a penetrating trauma to the temporal bone. The clinical manifestations of ECA‐PA can range from self‐limiting to life threatening. Our patient presented with massive otorrhagia requiring an acute control with an EAC Merocel packing. While otorrhagia is a well‐known symptom, to our knowledge, little is known about the adequate management in acute uncontrollable situations. In our experience, the anterior Merocel nasal packing has an expansile potential to create a seal in the EAC and a large surface area for absorbing blood. Additionally, the mastoid dressing was essential to maintain pressure on the area while preventing the packing from dislodging. Together, these measures temporized the situation, while the patient was transported to the IR suite. Angiographic evaluation of patients with suspected vascular injuries based on the location, type of injury, and presenting symptoms is critical for the early identification of a potentially fatal vascular injury.

The treatment modalities for massive otorrhagia include open surgical intervention and endovascular approach.^2^, ^5^ Our decision for intervention was based on the acuity of the presentation with profuse ongoing bleeding, the comminuted nature and location of the skull fractures, and the increased risk of prolonged general anesthesia in this critically ill patient. The endovascular approach proved to be effective in obliterating the pseudoaneurysm and therefore controlling the hemorrhage.

## 4 CONCLUSION

Otorrhagia will continue to be a fairly common otolaryngology complaint mostly in the traumatic setting. The vast majority of the otorrhagia cases are self‐limited. Nevertheless, we implemented a novel approach, which achieved a successful tamponade in a case of severe life‐threatening otorrhagia. This approach can be performed by any otolaryngologist in the community using easily accessible otolaryngology materials such as a Merocel packing.

## CONFLICT OF INTEREST

None declared.

## AUTHOR CONTRIBUTIONS

GR, SL, and MG: were involved with patient management; LG, GR, and SL: reviewed the inherent literature; GR and EL: prepared the manuscript; SL: edited the manuscript; all authors approved the final version of the manuscript.

## 5 ETHICAL APPROVAL

This study does not require any ethical committee approval.
